# Red light-promoted skin barrier recovery: Spatiotemporal evaluation by transepidermal potential

**DOI:** 10.1371/journal.pone.0219198

**Published:** 2019-07-10

**Authors:** Yuina Abe, Hajime Konno, Shotaro Yoshida, Takeshi Yamauchi, Kenshi Yamasaki, Mitsuhiro Denda, Matsuhiko Nishizawa

**Affiliations:** 1 Department of Finemechanics, Graduate School of Engineering, Tohoku University, Aramaki Aoba, Sendai, Japan; 2 Department of Dermatology, Graduate School of Medicine, Tohoku University, Seiryo-machi, Sendai, Japan; 3 Shiseido Research Center, Fukuura, Kanazawa-ku, Yokohama, Japan; Massachusetts General Hospital, UNITED STATES

## Abstract

The light-promoted recovery of epidermal barrier of skin was evaluated by the associated recovery of transepidermal potential (TEP), the potential difference between the surface and dermis of skin, by using porcine skin samples. An accelerated recovery of TEP was observed by irradiation of red light with the irradiance of 40 mW/cm^2^ and a duration of > 10 min. The influence of the light stimulation to the surroundings (~ 20 mm) was also observed. The irradiations of blue and purple lights were ineffective in accelerating the barrier recovery. These characteristics of the light stimulation would be useful for the design of effective and safe phototherapy devices for skin. The present study proves that the TEP can serve as a spatiotemporal indicator of the epidermal barrier function.

## Introduction

Thermal and chemical actions of light stimulation have been utilized for medical and cosmetic treatment of skin. Various laser technologies have been applied for skin treatments such as skin rejuvenation and scar-removal [1,2] and for wound healing [3]. Furthermore, uses of LED on skin care shows promising results [4], in addition to promoting the recovery of damaged epidermal barrier [5]. Studies on photochemical responses of skin have revealed that the photo-activated mitochondrial function improved the morphology and proliferation of epidermal cells [6]. Interestingly, the expression of a photoreceptor-like proteins in epidermal cells has been reported [7]. Those studies suggest that the epidermal cells would transduce light stimuli to promote the recovery of barrier and other functions of skin.

The epidermal barrier function has been conventionally evaluated by the measurement of transepidermal water loss (TEWL) [8]. Although the noninvasive TEWL measurement is convenient, this method is easily influenced by the surrounding environment, hydration of skin surface, and perspiration. Recently, transepidermal potential (TEP) has been reported as a possible index of epidermal barrier function [9,10], showing a correlation with TEWL [9]. TEP is the potential difference generated across the epidermis (thickness, ~200μm) by ion-transport of epidermal cells; its magnitude is up to tens of mV depending on body part [11] and varies reflecting the status of the epidermal barrier function [9,10]. We have recently developed a TEP measuring probe with a microneedle salt bridge, which enables minimally-invasive local TEP measurement [12].

In this study, we first demonstrated that the TEP measurement is useful to evaluate the epidermal barrier recovery promoted by irradiation of red light by using porcine skin samples. Then, the influence of light stimuli parameters (wave length, irradiation intensity, duration) were studied for optimization of light treatment condition. In addition, by taking advantage of the spatial resolution of TEP measurement, the spatial influence of irradiation was assessed for the first time. Two types of TEP measurement systems were applied: the conventional one using tubular salt bridges for measurement of the potential difference between the top surface of a skin sample and its base (dermis) immersed in a saline solution, and a probe-type system with a fine-needle salt bridge [12] for direct measurement of TEP generated at a targeted local point of the skin.

## Materials and methods

### Skin sample

Porcine abdominal skin with epidermis, dermis, and hypodermis with thickness of ~5 mm (Landrace swine, 6-month-old, castrated males, not pigmented, DARD Corp.) was transported to the laboratory on ice at ~0°C without freezing, stored in a refrigerator at 4°C before experiments, and used at room temperature. All the samples were used for experiments within 7 days after extraction, which were not expected to affect barrier healing and integrity since the refrigeration at 4°C was reported to reduce cell metabolism and tissue damage [13]. During experiments, the bottom surface of skin samples were immersed in Ringer’s solution to avoid drying. For disruption of the epidermal barrier, the outer surface of the sample was gently wiped with a cotton soaked with acetone (Wako Pure Chemical Industries, Ltd.) to remove lipids in the stratum corneum as previously reported [9, 12]. The barrier function of the stratum corneum is maintained by the structure consisted of cornified keratinocytes and lipids, and the acetone treatment causes loss of lipids that fill the intercellular spaces, which allows water to pass through, that is, the barrier function is lost [14]. Five sections were used for each experiment.

### Conventional TEP measurement

**Fig 1:**
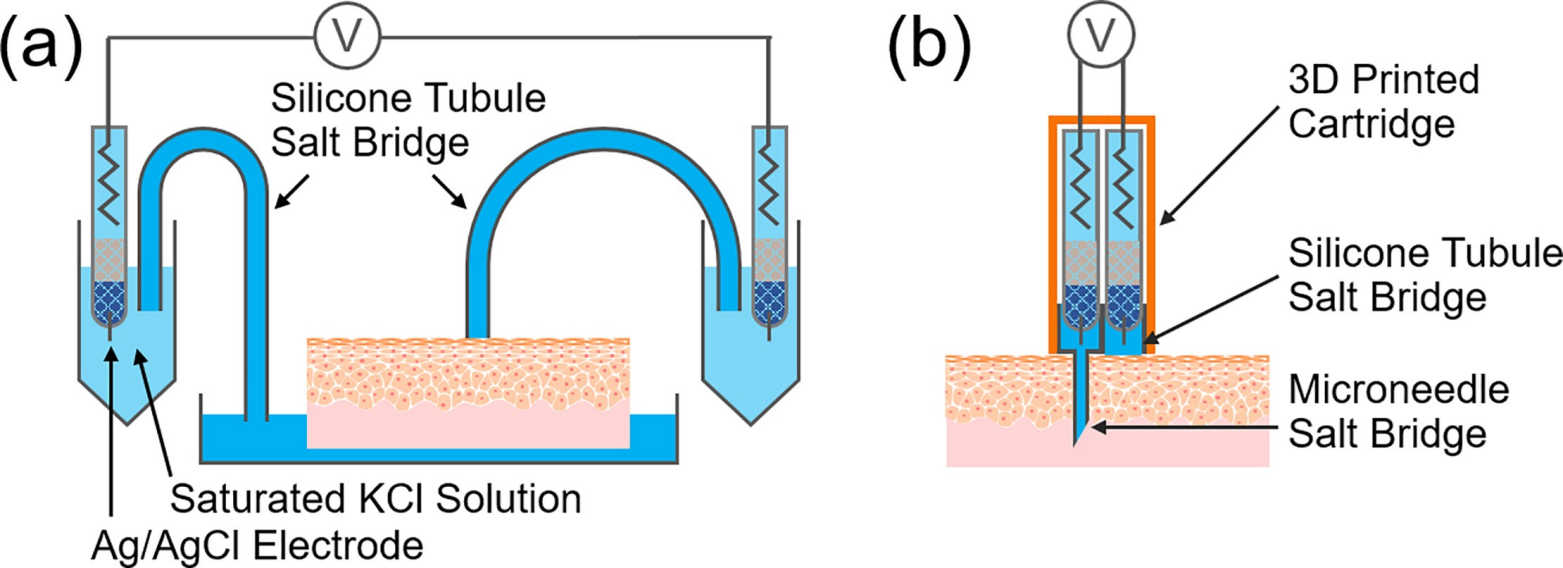
Schematic diagrams of TEP measurement systems. (a) Conventional system with silicone tubule salt bridges to measure the potential difference between the top surface of the skin sample and its base (dermis) immersed in a saline solution. (b) The fine needle-based probe system to directly measure the potential difference across epidermis at a targeted local point of the skin.

The entire system was assembled as shown in **Fig 1A**, according to the previous study [12,15]. Briefly, two Ag/AgCl electrodes were immersed in a saturated KCl solution and connected to the sample via tubular salt bridges using silicone rubber tubules (outer diameter 5 mm, inner diameter 3 mm) filled with 2 wt% agarose in Ringer’s solution. One of the tubular salt bridge was connected to the outer surface of the sample, while another was connected to the bottom of the sample via Ringer’s solution in which the skin sample was immersed. TEP was measured by a voltmeter (ALS 7082E, BAS Inc., operated in a voltmeter mode, 10 MΩ of input impedance).

### Probe-type TEP measurement

The measuring probe with a microneedle salt bridge which was previously reported [12] was improved as shown in **Fig 1B**; a hydrophilized painless syringe needle (Nanopass 34G Terumo Corp.) and silicone rubber tubule (outer diameter 5 mm, inner diameter 3 mm) were filled with 2 wt% agarose in Ringer’s solution, and Ag/AgCl reference electrodes were assembled in a custom-designed cartridge printed from the 3D printer (X-ONE 2, Qidi Technology).

### Light irradiation

White LED light source (LA-HDF5010, HAYASHI-REPIC Co., Ltd.; driven with a constant current and emitted continuous light), light guide with a 3 mm diameter optical fiber bundle (LGB (C) 1–3 L 1000), and filters (R-60 φ 22.5, HAYASHI-REPIC Co., Ltd., YIF-BP460-495S and YIF-BP400-440S, SIGMAKOKI Co., Ltd.; transmission wavelength of each filter was as follows; 600 nm and above (red), 464–489 nm (blue), 403–436 nm (purple)) were combined and used as the red, blue and purple light source. The optical power meter sensor (Optical Power Meter 3664 and Optical Sensor 9743, HIOKI E.E. Corp.) was mounted 5 mm below the tip of the light guide and the irradiance was set at 26 or 40 mW/cm^2^. Irradiance of 40 mW/cm^2^ was the maximum output of this system, while irradiance of 26 mW/cm^2^ was set to fit its fluence in 1h (93.6 J/cm^2^) to an intermediate value between 24.0 J/cm^2^ (10 min irradiation of 40 mW/cm^2^) and 144 J/cm^2^ (1 h irradiation of 40 mW/cm^2^). The irradiance of blue light was set to 40 mW/cm^2^ (144 J/cm^2^ for 1 h) to make it equal to the red light, and the irradiance of purple light was set to its maximum, 21 mW/cm^2^ (75.6 J/cm^2^ for 1 h). On the irradiation of skin samples, the tip of the light guide was placed 5 mm above the sample surface, and the light slightly spread from the tip of the light guide until the intense light hit the area of ~8 mm diameter on the surface. Temperature of the skin surface during light irradiations was measured with a temperature sensor (SK-8900, Sato Keiryoki Mfg. Co. Ltd.).

## Results

### Stimulation with visible light of different wavelength

**Fig 2:**
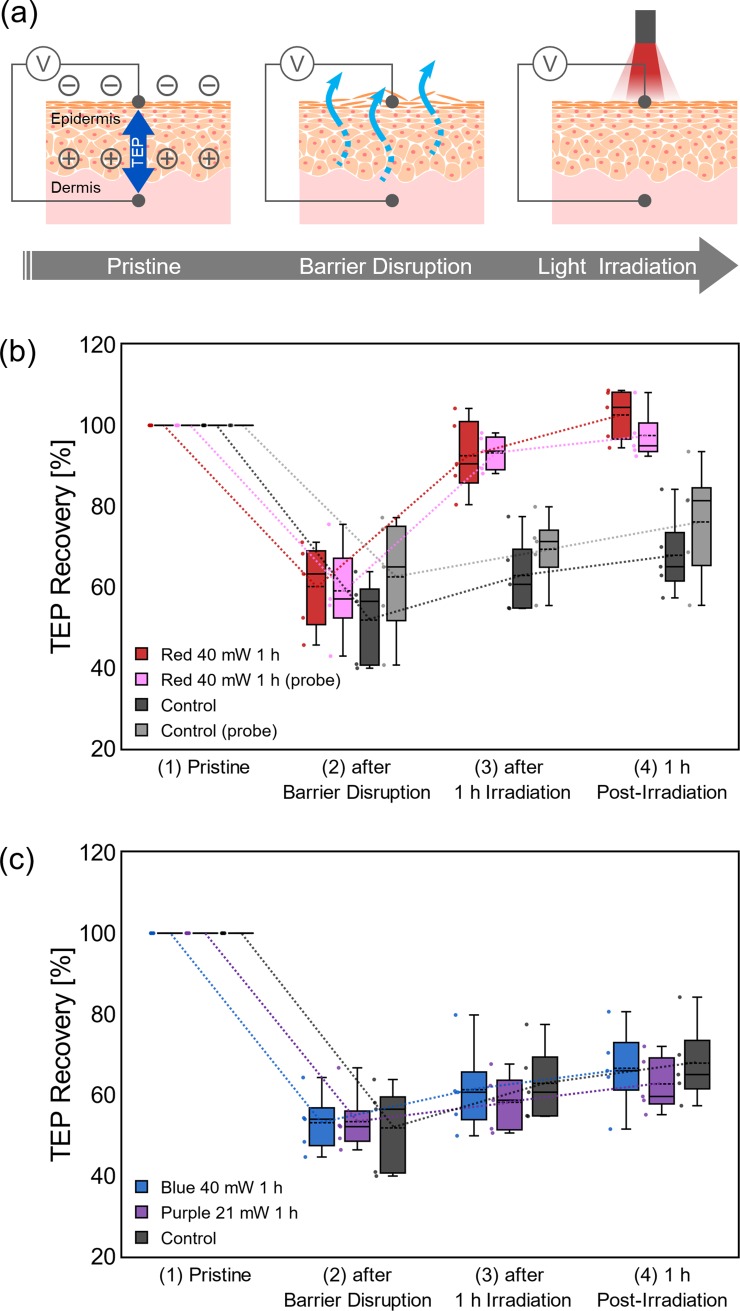
TEP changes by stimulation with visible light of different wavelength. (a) Schematic diagrams of evaluation of recovery of epidermal barrier at four stages: (1) pristine, (2) after disruption of barrier, (3) after 1h irradiation, and (4) 1 h post-irradiation. (b) TEP changes by red light irradiation (40 mW/cm^2^) with the original value of the pristine sample as 100%, measured by the conventional system (red bars) and the probe system (pink bars). TEP changes without light irradiation are also shown by black and gray bars as the control. (c) TEP changes measured for irradiations of blue/purple lights (40 and 21 mW/cm^2^). The number of samples for each experiment was 5. Lines and dashed lines in the bars represent median and mean, respectively.

As schematically illustrated in **Fig 2A**, in order to examine the skin barrier recovery promoted by red light irradiation, TEP of porcine skin sample were measured at each of the four states of a sample: (1) pristine, (2) after disruption of the barrier (20 mm x 20 mm), (3) after 1h of irradiation, (4) and 1h post-irradiation. The TEP values for the pristine samples were 4.11 mV~ 17.8 mV (the potentials at the subepidermal tissue against the surface of the skin reference) that was decreased when epidermal barrier was disrupted by wiping with acetone [12,16], following the removal of the lipid that filled the intercellular space between the cornified keratinocytes[14]. **Fig 2B** shows the TEP changes during the barrier disruption and light irradiation, with the original value of the pristine sample as 100%. When the red light of 40 mW/cm^2^ was irradiated for 1 h (144 J/cm^2^), TEP recovered almost to its original level (red bars), and the recovery was obviously faster than the control (black bars). The temperature change at the sample surface during the red light irradiation was negligibly small (< 1°C), ensuring the observed acceleration of TEP recovery is proceeded by photochemical action of the light, not by a thermal action. The features of TEP recovery measured by the probe system (pink and gray bars) were almost the same as the conventional system. Though the subsequent experiments in this work were carried out by the conventional system, the probe system is necessary for future animal experiments and applications to human subjects with minimal invasion.

**Fig 2C** shows the TEP during the 1h irradiations of 40 or 21 mW/cm^2^ blue light or purple light (144 or 75.6 J/cm^2^). In contrast to the clear effect of the red light irradiations, the recovery during the blue / purple irradiations was about the same or rather slower than the control (without irradiation). These results are in agreement with the previous study based on TEWL measurement for a hairless mouse [5], in which an irradiation of blue light showed an inhibitory effect to delay the recovery of the barrier function. Penetration depth of the blue and violet light thorough skin are 0.3 ~ 0.4 mm [17], and the depth of red light is deeper than the blue and violet light. The depths were not relevant to the porcine barrier recovery process since they were deeper than the thickness of epidermis (mean ~0.1 mm) (**S1 Fig**). In fact, direct irradiation of the blue light to the exposed dermis did not show any significant effect on the TEP recovery (**S2 Fig**). Red light that penetrates deeper into the skin appears superior at least based on our experiment. Therefore, it could be concluded that the wavelength of the red light (> 600 nm) is important for recovery of skin barrier.

### Red light stimulation with different irradiance and duration

**Fig 3:**
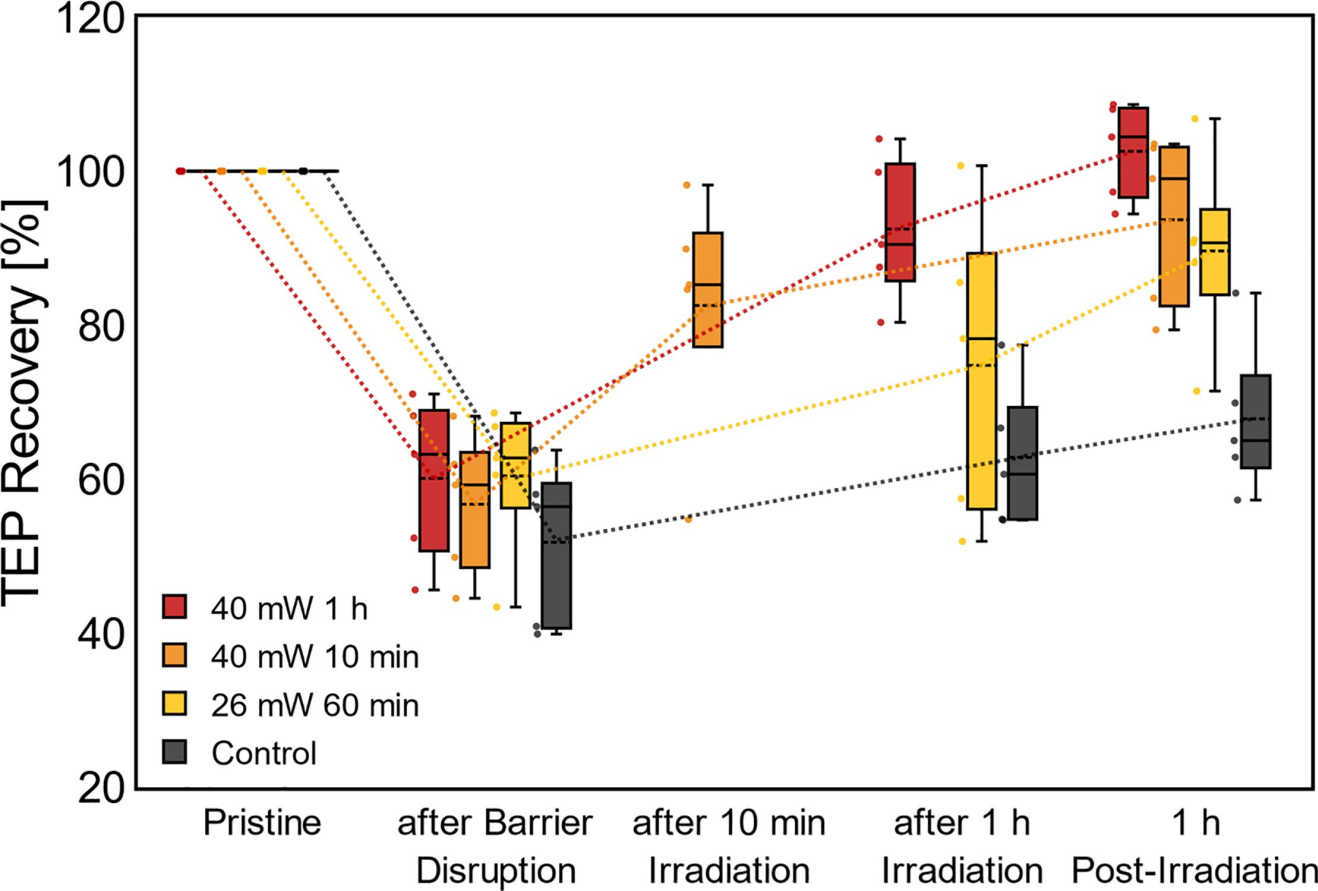
TEP changes measured by the conventional system during red light irradiation with different irradiance and duration. 40mW/1h (red bars), 40mW/10min (orange bars), 26mW/1h (yellow bars) and control without irradiation (black bars). The sample number for each experiment was 5. Lines and dashed lines in the bars represent median and mean, respectively.

In the above experiments, we set the standard irradiance to 40 mW/cm^2^, which is within the normal range for a safe LED treatment of skin [4] It was also the maximum output of the light source we used. Here, the influence of irradiance was studied by using a weaker irradiance of 26 mW/cm^2^ (93.6 J/cm^2^), as shown in **Fig 3** with yellow bars. In contrast to the clear recovery obtained with 40 mW/cm^2^ (red bars), the TEP value with 26 mW/cm^2^ irradiation was not recovered to the original level even after 1h post- irradiation, and the variation of data was larger. From these results, 40 mW/cm^2^ irradiance was confirmed to be sufficient to obtain a remarkable effect in the promotion of barrier recovery, while 26 mW/cm^2^ irradiance was not. As far as we know, this is the first study of the light-promoted recovery of epidermal barrier with controlled irradiance and duration, which has been achieved by taking advantage of the quick, local TEP measurement systems that we developed.

Next, the duration of the 40 mW/cm^2^ irradiation was shortened to 10 min (24.0 J/cm^2^, orange bars in **Fig 3**) from the standard 1h that was set according to the previous report [5]. It was found that the TEP value recovered to the original level similarly to the 1h irradiation within 10 min (similarly, within 20 min and 30 min, **S3 Fig**). Taking together with the above insufficient effect of the 26 mW/cm^2^ irradiance for even 1h duration, there seems to be a threshold of irradiance to activate epidermis for obvious recovery of skin barrier. Cytotoxicity has been reported to occur at high fluence (high light intensity and long time exposure) [18], and it is desirable to reduce the exposure time to obtain a therapeutic effect more safely.

### Spatial influence of red light stimulation

**Fig 4:**
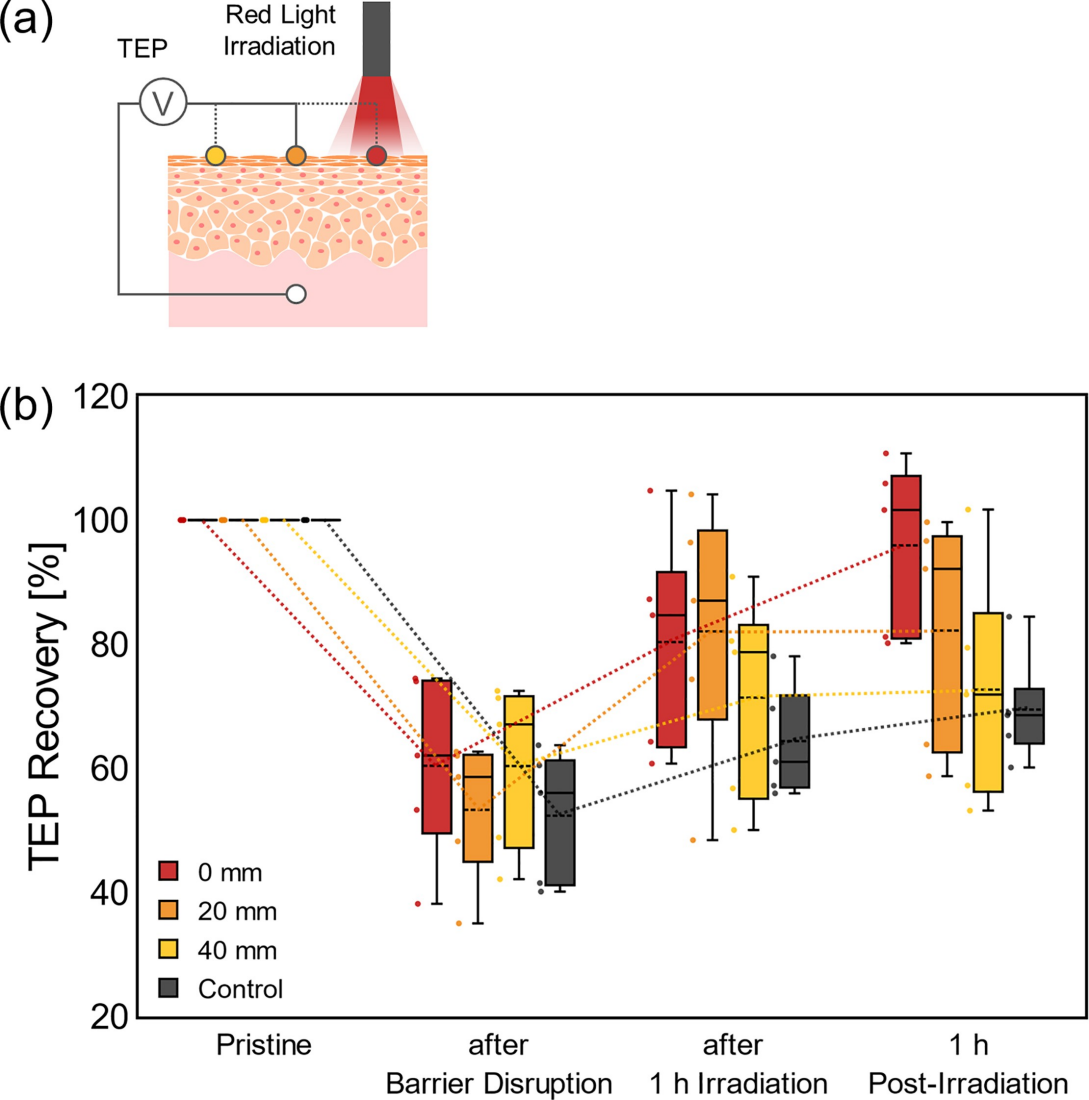
Spatial influence of red light stimulation. (a) Schematic diagrams of TEP measurements to evaluate spatial influence of the effect of red light stimulation. (b) TEP changes by red light irradiation (40 mW/cm^2^) measured at the distance of 0 mm (red), 20 mm (orange) and 40 mm (yellow) apart from the irradiated point. The number of samples for each experiment was 5. Lines and dashed lines in the bars represent median and mean, respectively.

The TEP measurements were conducted at the points apart from the red light irradiation (40 mW/cm^2^ for 1h), as shown in **Fig 4**. The epidermal barrier was disrupted by wiping it with acetone, and TEPs were measured at the distance of 0 mm (red), 20 mm (orange) and 40 mm (yellow) apart from the irradiated point. Although the recovery of TEP was not obvious compared with those in **Figs 2B and 3** (disruption area, ca. 4 cm^2^) due to skin damage during the barrier disruption of wider area (ca. 12 cm^2^), some promoting effect in TEP recovery could be observed even at the point 20 mm apart. Since the irradiance measured at the point of 20 mm was extremely small at the level of μW/cm^2^, the results in **Fig 4** indicate that the physiological effect of red light stimulation could spread to some distance around the irradiated point.

## Discussion

Here, by using TEP values as a novel indicator, it was clearly examined that the red light irradiation promotes the recovery of epidermal barrier (**Fig 2B**). Since the temperature at the skin surface was not changed by the irradiation, the reasons to the observed barrier recovery should not be thermal phenomena. The characteristics of TEP change during the light irradiation were almost the same for both conventional and probe-type systems. The latter system directly measured the local TEP by setting the potential reference right under the measuring point, minimizing the influence of surrounding tissue like dermis and hypodermis. From the similarity between the results of these two systems, the direct effect of the dermis on the measured TEP in the present experiments is negligibly small, although dermal fibroblasts have been reported to respond to light stimuli [6]. Since the promotion effect was not observed by the blue / purple irradiations (**Fig 2C**), it could be concluded that the wavelength of the red light (> 600 nm) is beneficial for the recovery of skin barriers. From the studies of stimuli parameters (**Fig 3**) by varying irradiance (40 mW/cm^2^ and 26 mW/cm^2^) and the duration (1h and 10 min), no simple reciprocity was found between these two parameters. The stimulatory effect of a weaker irradiance (26 mW/cm^2^) was not clear even with a long duration of exposure (1h). There seems to be a threshold of irradiance to activate epidermis for obvious recovery of the skin barrier. Similar observation has been reported for light therapy of wound [19], in which a long exposure at low irradiance was ineffective in accelerating wound healing. The fluence of light stimulation has been reported to have a therapeutic window, out of which it showed an inhibitory effect. The threshold suggested from our result is important to obtain the therapeutic effect safely. Lastly, the lateral influence of the light stimulation was studied by taking advantage of the spatial resolution of TEP measurement (**Fig 4B**). It was found that the physiological effect of the red light stimulation could spread some distance (~20 mm) away from the irradiated point. Since the light irradiance was weakened in an inverse square of the distance and below the threshold, this “spread” effect was unlikely to be induced by the light directly. Rather, it seemed to be an effect propagated through other pathways, such as signaling between epidermal keratinocytes which were reported to induce calcium wave [20]. From the therapeutic point of view, the results obtained here indicate that a sufficient irradiance (~40 mW/cm^2^) exceeding the threshold can shorten the time required for light therapy for skin, and that the device for skin stimulation can be flexibly designed assuming the spread of the stimulatory effect to its surroundings. As the therapeutic effect of the red light irradiation has also been reported with a clinically used, standardized method TEWL [5], and correlation of TEWL and TEP as the barrier indicators has been confirmed [9] (which was also measured in **S4 Fig** and **S1 Protocol**), the light-induced accelerated barrier recovery measured by TEP in this study should be clinically plausible. The TEP measurement will be clinically useful for spatiotemporal testing of skin barrier condition in a more convenient manner than conventional methods.

## Conclusions

In this study, we demonstrated the light-promoted recovery of epidermal barrier by spatiotemporal TEP measurements. The effect of red light irradiation to the barrier recovery [5] was confirmed to be correlated to the recovery of the original TEP value. We studied the stimuli parameters and found conditions for remarkable recovery to be irradiance at ~40 mW/cm^2^ and duration of 10 min or more. In addition, the light-promoted recovery was found to spread from the light-irradiated point to some distance (~20mm). These characteristics of light treatment should be useful to optimize the design of a light stimulation device for effective, convenient, and safe skin treatments.

## Supporting information

S1 Fig Epidermal thickness of porcine skin sample. The thickness was measured at randomly picked points from the HE tissue section images. 5 images, and 3 points per image were used to find the mean value. (a)Epidermal thickness. The point with bar represents mean and standard error respectively. The other points represent individual data points. (b)Representative image of the measured sample. 3 lines indicate the measured points.
(TIF)

S2 Fig TEP changes by stimulation by blue light. TEP measurement during blue light irradiation with different depth. (a) Schematic diagram for experimental system. The blue light was irradiated right on the surface of the epidermis or the dermis (the epidermis was removed by surgical scissors). To avoid the influence of the wound of epidermis-removal, measuring point was taken 20 mm away from the irradiation point. (b)TEP recovery (rate to initial value) in samples which were irradiated with blue light. Light blue: light-irradiated right on the epidermal surface, Blue: light-irradiated on the dermal surface, and Black: control (same as “Control” in **Fig 2B**). Lines and dashed lines in the boxes represent median and mean respectively.
(TIF)

S3 Fig TEP changes by stimulation by red light. TEP measurement during red light irradiation with different duration. Orange: 40mW/30min, Yellow: 40mW/20min, Black: control without irradiation (same as “Control” in **Fig 2B**). The sample number for each experiment was 5. Lines and dashed lines in the bars represent median and mean, respectively.
(TIF)

S4 Fig Chronological, simultaneous measurement and correlation between TEP and TEWL. TEP and TEWL during stepwise barrier destruction were measured. TEP was measured by the probe, while TEWL was measured by Tewameter (Courage + Khazaka electronic GmbH). The participant was healthy adult female aged >20 at informed consent. The Ethical Committee of the Graduate School of Engineering, Tohoku University approved the experimental procedures described herein.
(TIF)

S1 Protocol TEP and TEWL measurement of human participant. To confirm the correlation between TEP and clinically standardized index, TEWL, their parallel measurement was conducted. TEP was measured by the probe, while TEWL was measured by Tewameter (Courage + Khazaka electronic GmbH). The participant was healthy adult female aged >20. A flat point on the posterior forearm which hair was removed was marked as a measurement point, and then TEWL and the TEP of pristine skin were measured. Subsequently, adhesive tape (Nichiban Co., Ltd., Tokyo, Japan) was adhered on the point and peeled off repeatedly to reduce the barrier integrity gradually (barrier disruption method known as tape stripping). TEWL and TEP were measured at the following point; tape stripping of 1, 4, 7, 10 times. All procedures performed in studies involving human participants were in accordance with the standards of the Ethics Committee of Graduate School of Engineering, Tohoku University (16A-5) and with the 1964 Helsinki declaration and its later amendments. Before experiments, the purpose of this study was explained to subjects who signed the university institutional approved informed consent.
(TIFF)

S1 Table Data set for drawing figures. (XLSX)
