# Supplementary figures and images for: Red light-promoted skin barrier recovery: Spatiotemporal evaluation by transepidermal potential
Source: PLoS One. 2019 Jul 10;14(7):e0219198. doi: 10.1371/journal.pone.0219198 (PMC6620005; doi:10.1371/journal.pone.0219198)

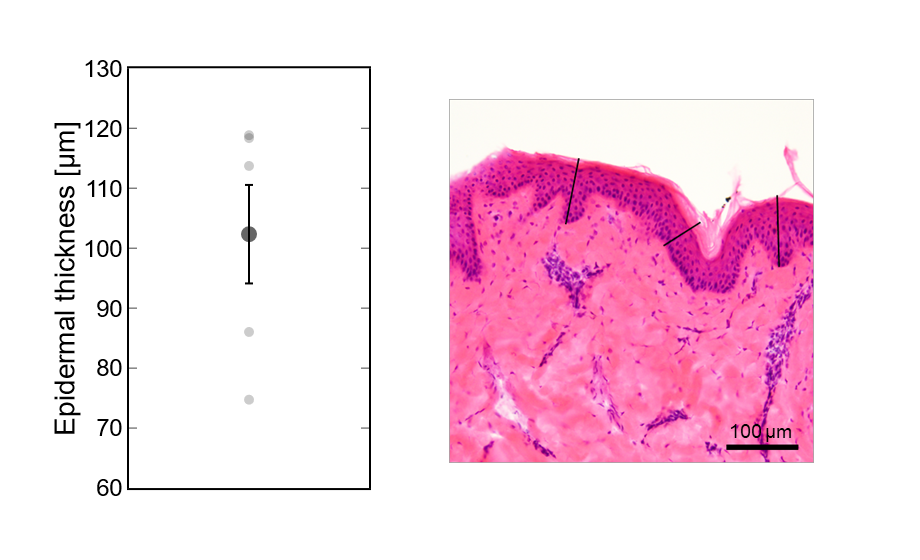

Supplement: S1 Fig — The thickness was measured at randomly picked points from the HE tissue section images. 5 images, and 3 points per image were used to find the mean value. (a)Epidermal thickness. The point with bar represents mean and standard error respectively. The other points represent individual data points. (b)Representative image of the measured sample. 3 lines indicate the measured points. (TIF) [file pone.0219198.s001.tif]

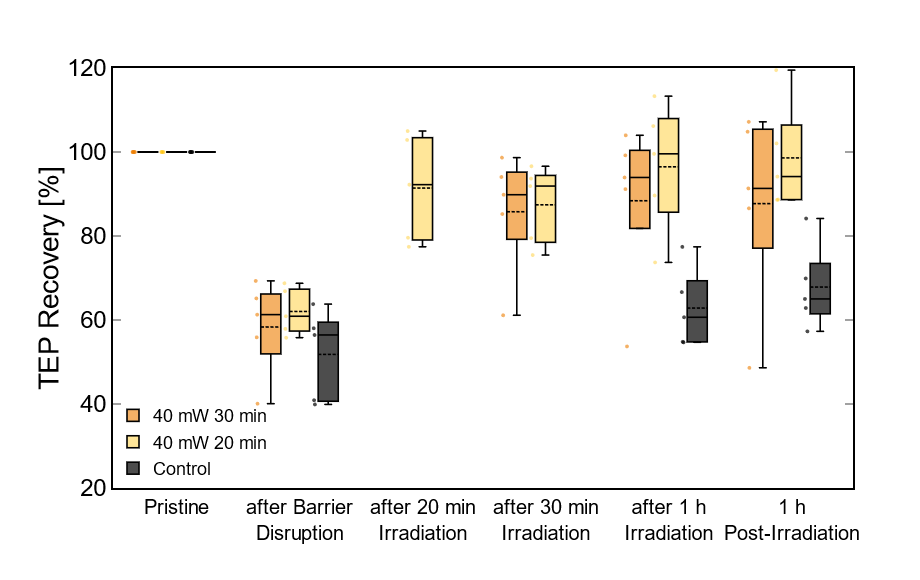

Supplement: S2 Fig — TEP measurement during blue light irradiation with different depth. (a) Schematic diagram for experimental system. The blue light was irradiated right on the surface of the epidermis or the dermis (the epidermis was removed by surgical scissors). To avoid the influence of the wound of epidermis-removal, measuring point was taken 20 mm away from the irradiation point. (b)TEP recovery (rate to initial value) in samples which were irradiated with blue light. Light blue: light-irradiated right on the epidermal surface, Blue: light-irradiated on the dermal surface, and Black: control (same as “Control” in Fig 2B). Lines and dashed lines in the boxes represent median and mean respectively. (TIF) [file pone.0219198.s002.tif]

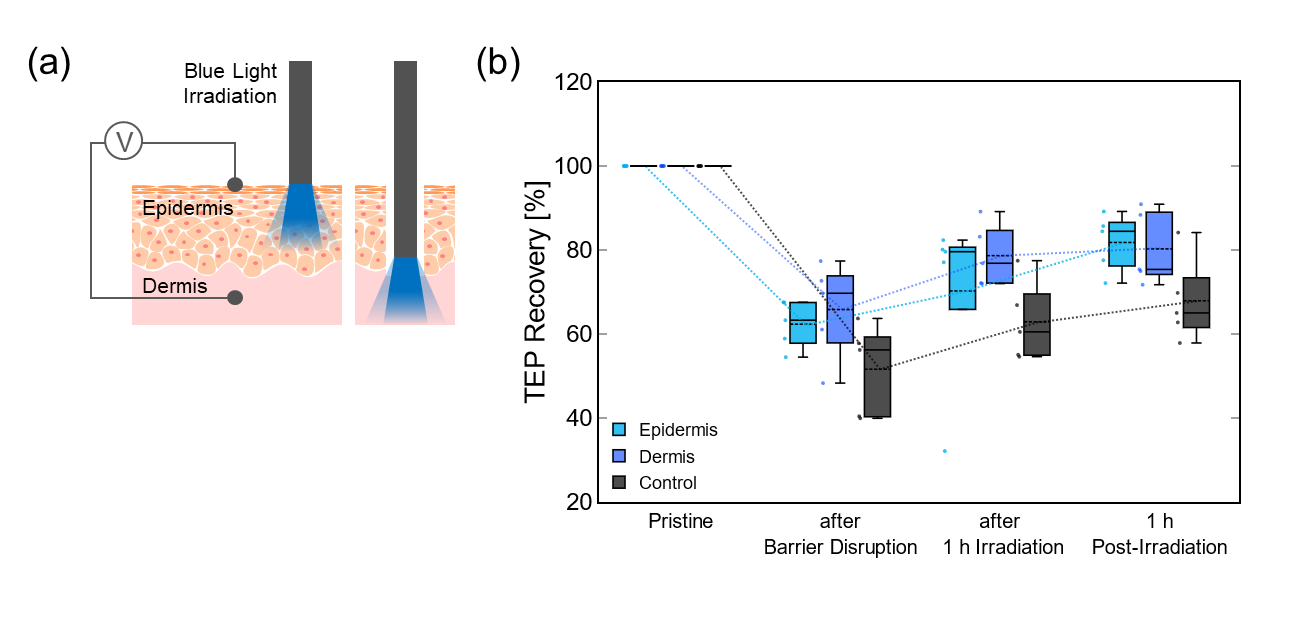

Supplement: S3 Fig — TEP measurement during red light irradiation with different duration. Orange: 40mW/30min, Yellow: 40mW/20min, Black: control without irradiation (same as “Control” in Fig 2B). The sample number for each experiment was 5. Lines and dashed lines in the bars represent median and mean, respectively. (TIF) [file pone.0219198.s003.tif]

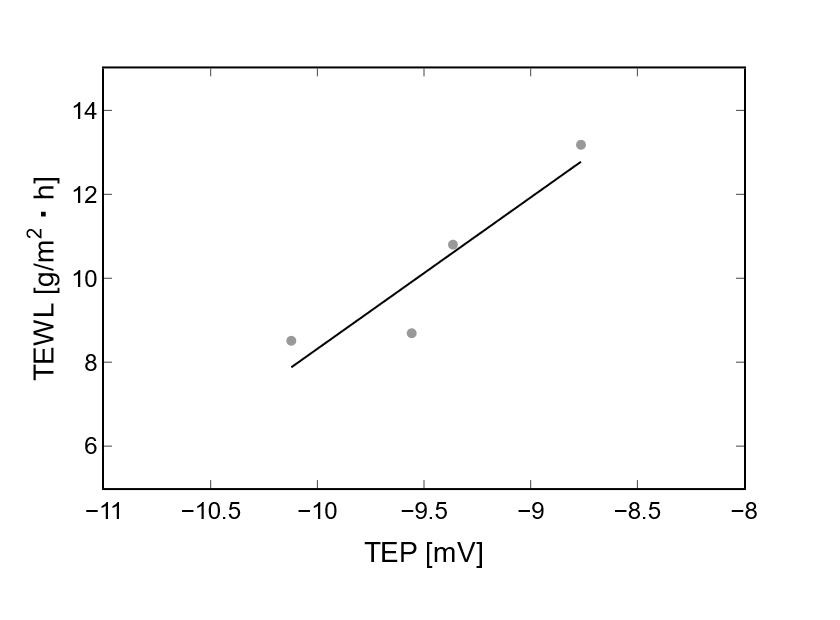

Supplement: S4 Fig — TEP and TEWL during stepwise barrier destruction were measured. TEP was measured by the probe, while TEWL was measured by Tewameter (Courage + Khazaka electronic GmbH). The participant was healthy adult female aged >20 at informed consent. The Ethical Committee of the Graduate School of Engineering, Tohoku University approved the experimental procedures described herein. (TIF) [file pone.0219198.s004.tif]
